# Physical Activity, Psychological Resilience, and Non-Suicidal Self-Injury Among Male College Students: A Cross-Sectional Study

**DOI:** 10.3390/healthcare14193218

**Published:** 2026-09-29

**Authors:** Chang Hu, Wen Zhang, Muxiang Che, Joston Gary

**Affiliations:** 1 School of Physical Education, Hunan First Normal University, Changsha 410205, China; huchang@hnfnu.edu.cn; 2 School of Physical Education, Jiangxi Normal University, Nanchang 330022, China; zhangwen@jxnu.edu.cn; 3 School of Physical Education, Guiyang University, Guiyang 550005, China; chemx_0225@gyu.edu.cn; 4 Department of Management and Engineering, Linköping University, SE-581 83 Linköping, Sweden; 5 Department of Economics, Management, Industrial Engineering and Tourism, University of Aveiro, 3810-193 Aveiro, Portugal; 6 School of Economics, Management and Political Science, University of Minho, 4710-057 Braga, Portugal

**Keywords:** exercise volume, psychological resilience, non-suicidal self-injury, male college students, indirect association, latent profile analysis, cross-sectional study

## Abstract

**Background/Objectives**: Non-suicidal self-injury (NSSI) is an important mental health concern among college students, yet evidence regarding behavioral and psychological correlates of NSSI among male students remains limited. This study examined the cross-sectional associations among physical activity (PA), psychological resilience (PR), and NSSI in male college students and explored heterogeneity in joint PA–PR patterns. **Methods**: A cross-sectional survey was conducted among 2567 male college students from one university in Jiangxi Province, China. PA, PR, and NSSI were assessed using the Physical Activity Rating Scale-3, the 10-item Connor–Davidson Resilience Scale, and 10 behavioral-frequency items from the Ottawa Self-Injury Inventory, respectively. Pearson correlations, covariate-adjusted regression analyses, bootstrap analysis of the statistical indirect association, sensitivity analyses, and latent profile analysis (LPA) were performed. **Results**: PA was positively associated with PR (r = 0.627, *p* < 0.001), whereas PA and PR were negatively associated with NSSI (r = −0.259 and −0.292, respectively; both *p* < 0.001). After adjustment for all measured demographic characteristics, PR statistically accounted for part of the negative association between PA and NSSI (indirect effect = −0.133, 95% bootstrap CI [−0.164, −0.102]). The NSSI model explained 9.8% of the variance. Past-year NSSI was reported by 35.41% of participants. LPA identified two predominantly level-based profiles: Low PA–Low PR and High PA–High PR. Participants in the Low PA–Low PR profile reported significantly higher NSSI scores than those in the High PA–High PR profile. **Conclusions**: Higher PA and PR were associated with lower NSSI among male college students, and PR statistically accounted for part of the cross-sectional PA–NSSI association. The two PA–PR profiles also differed in NSSI levels. These findings indicate that PA and PR are relevant correlates of NSSI, although their temporal and causal relationships require further investigation.

## 1. Introduction

Non-suicidal self-injury (NSSI), defined as the deliberate destruction of one’s own body tissue without suicidal intent, represents an important mental-health concern during adolescence and emerging adulthood [1,2]. Although NSSI commonly begins during adolescence, it does not necessarily disappear after the transition to university; pooled evidence indicates that self-injury remains clinically relevant among young adults, including university populations [3,4]. Beyond its occurrence, functional models of NSSI emphasize that the behavior may serve intrapersonal and interpersonal functions, particularly the regulation of intense negative affect and the management of aversive internal experiences [5,6,7]. The experiential avoidance model similarly proposes that self-injury can be maintained because it provides short-term escape from unwanted emotional states. These perspectives suggest that understanding NSSI requires moving beyond the identification of psychopathological risk factors toward examining the behavioral and psychological resources that may correspond with more adaptive responses to stress [8,9,10].

The university period provides an important context in which to examine such resources. Students encounter increasing academic demands, changing social networks, greater personal autonomy, and concerns regarding future employment, while their established coping resources may simultaneously be challenged. Male college students warrant particular attention, not because NSSI can be assumed to be more prevalent among men, but because the psychosocial context in which psychological difficulties are expressed and managed may differ by gender [11]. Evidence regarding sex differences in NSSI is heterogeneous across populations, whereas research on psychological help-seeking consistently suggests that men are less likely to seek professional support and that conformity to norms emphasizing emotional control and self-reliance may further inhibit help-seeking [12,13]. Consequently, identifying observable or modifiable health behaviors and positive psychological resources associated with NSSI may be particularly informative in male university populations, in whom psychological distress may not always translate into formal help-seeking [14,15].

Physical activity (PA) represents one such potentially modifiable behavioral factor. Regular exercise provides repeated experiences of effort, goal attainment, recovery, social interaction, and affective regulation, all of which may be relevant to how individuals respond to psychological strain [16,17]. Initial evidence suggests an inverse association between PA and NSSI. Boone and Brausch found that lower physical activity was associated with greater NSSI frequency in a sample of adolescents and young adults, whereas a recent systematic review of longitudinal studies concluded that low level of physical activity was associated with greater subsequent NSSI risk [18]. However, the available evidence remains heavily concentrated in adolescent samples; in the longitudinal review, participants were predominantly adolescents and approximately three quarters were female. Thus, whether exercise behavior is similarly associated with NSSI among male university students in emerging adulthood remains insufficiently characterized. Moreover, existing studies have generally treated PA as a correlate of NSSI rather than examining how it operates alongside positive psychological resources [19].

Psychological resilience (PR) may provide an important theoretical bridge between these constructs [20]. Conservation of Resources theory proposes that individuals strive to acquire, maintain, and protect resources that facilitate adaptation under conditions of stress [21]. From this perspective, PA can be conceptualized cautiously as a health behavior that may coincide with opportunities for resource gain through mastery experiences, perceived competence, structured coping, and social engagement, whereas PR represents a personal psychological resource reflecting the capacity to maintain or regain adaptive functioning under adversity. Consistent with this account, a recent systematic review and meta-analysis found a reliable positive association between PA and PR among young students [22]. Separately, a systematic review of 17 studies involving more than 12,000 participants found that higher resilience was consistently associated with lower NSSI and that resilience frequently functioned as a protective, mediating, or moderating factor in the context of stress and self-injury [23]. Recent research among college students similarly indicates that resilience is relevant to the association between stress-related experiences and NSSI [24,25].

The functional account of NSSI and Conservation of Resources theory provide complementary explanations for the present study. Functional models explain why NSSI may emerge as a maladaptive strategy for regulating distress, whereas the resource perspective directs attention to behavioral and psychological assets that may correspond with alternative patterns of adaptation. Within this framework, greater self-reported exercise volume may be associated with greater PR, and both may be associated with lower NSSI. Importantly, because these constructs were assessed cross-sectionally in the present study, this framework is used to organize expected associations rather than to establish temporal mediation or causal pathways. Within this framework, PR is examined as a psychological resource that may help explain the observed association between PA and NSSI.

A second limitation of the existing literature concerns its predominant reliance on variable-centered analyses. Correlation, regression, and indirect-effect models estimate average associations and implicitly assume that the same general pattern adequately describes the entire sample. Yet students may differ substantially in the way behavioral and psychological resources co-occur: relatively low exercise volume and low resilience may occur together in some individuals, whereas other configurations may also be present. Person-centered approaches such as latent profile analysis (LPA) can complement variable-centered analyses by identifying naturally occurring configurations across continuous indicators [26]. Previous NSSI research has demonstrated the usefulness of person-centered methods for detecting heterogeneity in psychological and behavioral characteristics, and a recent study has begun to integrate PA and PR using variable- and person-centered approaches in adolescents [27,28,29,30]. However, whether comparable PA–PR configurations are evident among male college students—and whether these configurations differ in NSSI—remains unclear. This represents an important extension because developmental context, autonomy, stress exposure, and patterns of help-seeking differ between adolescence and emerging adulthood.

Accordingly, the present study extends previous research in three respects. First, it focuses specifically on male college students, a comparatively understudied population in the PA–PR–NSSI literature, without assuming that men have a higher prevalence of NSSI. Second, it integrates a potentially modifiable health behavior and a positive psychological resource within a theoretically informed framework, testing whether PR statistically accounts for part of the cross-sectional association between self-reported exercise volume and NSSI. Third, it combines variable-centered analyses with LPA to examine whether distinct joint configurations of PA and PR can be identified and whether NSSI differs across these profiles. This complementary strategy allows the study to address both how variables are associated on average and whether meaningful heterogeneity exists between individuals. Given the cross-sectional design, all hypotheses concern associations.

### 1.1. Physical Activity and Non-Suicidal Self-Injury

PA refers to bodily movement produced by skeletal muscles that requires energy expenditure, including daily movement, exercise, and organized sport [31]. Its potential relevance to NSSI can be understood in relation to the emotional and behavioral functions of self-injury. Functional accounts of NSSI suggest that self-injury may occur, in part, as a maladaptive strategy for managing intense negative affect or aversive internal experiences. In contrast, greater engagement in PA has been associated with lower levels of depression, anxiety, and perceived stress [32,33,34], as well as with more positive affect, greater perceived competence, social engagement, and more adaptive emotion regulation [35,36]. These characteristics may be relevant to NSSI because they correspond with alternative behavioral and psychological resources for coping with distress.

Empirical evidence is generally consistent with this possibility. Higher levels of PA have been associated with lower NSSI among adolescents and young adults [37,38], whereas lower PA and greater sedentary behavior have been linked to higher levels of NSSI and related risk behaviors [39,40,41]. Emotion regulation may be one mechanism underlying these associations, given that PA is related to lower negative affect and more adaptive emotional responding, much of the existing evidence has been derived from adolescent or mixed-gender samples, and relatively little is known about whether a similar pattern is evident among male college students in emerging adulthood.

The available evidence, therefore, supports testing an inverse association between self-reported exercise volume and NSSI, but it does not establish a causal protective effect. Exercise participation may correspond with social contact, structured routines, affect regulation, and perceived competence, while reverse or shared-cause explanations are also possible. Thus, the following hypotheses are put forward.

**H1.**  *Physical activity is negatively associated with NSSI among male college students.*

### 1.2. Psychological Resilience in the Association Between Physical Activity and NSSI

PR refers to the capacity to maintain or regain adaptive functioning in the face of stress and adversity [42]. From a stress–adaptation perspective, individuals exposed to similar environmental demands may respond differently depending on the psychological resources available to them [43]. PR is therefore particularly relevant to NSSI because it reflects the capacity to tolerate, recover from, and adapt to distress without relying on maladaptive coping behaviors.

Consistent with this view, higher PR has been associated with lower levels of depression, anxiety, and behavioral difficulties [44,45,46], whereas lower PR is related to greater emotion-regulation difficulties and engagement in risk behaviors [47,48]. Similar patterns have been reported for NSSI: individuals with greater resilience tend to report lower levels or likelihood of self-injury [23,49,50]. In addition, PR has been shown to statistically account for part of the associations between adverse experiences, including negative life events, peer victimization, and emotional difficulties, and NSSI [51,52]. These findings suggest that PR may represent an important psychological resource associated with how individuals respond to stress-related vulnerability.

The potential link between PA and PR can be further understood through Conservation of Resources theory, which proposes that individuals strive to acquire, preserve, and protect resources that support adaptation under stress [21]. Exercise participation is often accompanied by experiences of mastery, goal attainment, recovery, perceived competence, and social engagement, all of which may correspond with stronger psychological resources [53,54]. Within this framework, PA can be conceptualized as a health behavior associated with opportunities for resource development, whereas PR represents a personal resource that may be relevant to emotional and behavioral regulation [55,56]. Importantly, given the cross-sectional nature of the present study, these relationships are interpreted as patterns of association rather than as evidence that PA produces increases in PR.

Prior research and the stress-adaptation and conservation-of-resources perspectives suggest that PA may be positively associated with psychological resilience, whereas psychological resilience may be negatively associated with NSSI. Therefore, the following hypothesis is put forward.

**H2.**  *Psychological resilience statistically accounts for part of the negative association between PA and NSSI among male college students.*

### 1.3. Joint Profiles of Physical Activity and Psychological Resilience

Variable-centered analyses estimate average associations among PA, PR, and NSSI across the sample, but such averages may obscure meaningful heterogeneity between individuals. Students with similar overall levels of NSSI may differ substantially in the behavioral and psychological resources they possess, and the same average association can arise from different within-person configurations of PA and PR. A person-centered perspective is therefore useful for examining whether distinct combinations of these resources coexist within the population.

PA and PR represent conceptually related but distinct forms of adaptive resources. PA reflects behavioral engagement, whereas PR reflects the psychological capacity to adapt to stress and adversity. Although greater PA may commonly co-occur with stronger PR, this correspondence need not be uniform across individuals. Some students may exhibit relatively high levels of both resources, others relatively low levels of both, and still others may display less concordant combinations. Identifying such patterns is important because different behavioral–psychological configurations may correspond with different levels of vulnerability to NSSI.

LPA provides a person-centered approach for identifying naturally occurring subgroups based on continuous indicators without imposing categories in advance [57,58]. In the present context, LPA complements the variable-centered analyses by addressing a different question: rather than asking only whether PA and PR are associated with NSSI on average, it examines whether students can be grouped according to distinct joint patterns of PA and PR and whether these groups differ in NSSI. This approach may therefore provide a more nuanced account of heterogeneity in behavioral and psychological resources among male college students. Accordingly, the present study examined whether distinct joint PA–PR profiles could be identified and whether NSSI levels differed across these profiles. Therefore, the following hypothesis is put forward.

**H3.**  *Distinct joint profiles of PA and psychological resilience can be identified among male college students, and NSSI levels differ across these profiles.*

### 1.4. Study Objectives

This study examined the cross-sectional associations among PA, psychological resilience, and NSSI in male college students from a single university in China. Variable-centered analyses were used to test the negative association between PA and NSSI proposed in H1 and to examine whether psychological resilience statistically accounted for part of this association, as specified in H2. All measured demographic characteristics were included as covariates. Person-centered analysis was then conducted to address H3 by identifying joint PA–psychological resilience profiles and examining differences in NSSI across these profiles. Given the cross-sectional design and single-university sample, the study was not intended to establish temporal ordering or causal mechanisms, nor to provide nationally representative prevalence estimates.

## 2. Materials and Methods

### 2.1. Participants

The present study reports a cross-sectional analysis of baseline data collected between July and August 2025 as part of a broader longitudinal project conducted at one university in Jiangxi Province, China. A single-site convenience sampling strategy was adopted to support cohort continuity and the feasibility of subsequent follow-up under relatively consistent institutional conditions. Participants were recruited through the university’s student network and online peer referral, including WeChat groups and personal networks. Although this strategy facilitated recruitment and future follow-up, it did not yield a probability-based sample. Accordingly, the sample should be regarded as institution-specific and not representative of all male university students in Jiangxi Province or China. All questionnaires were completed electronically.

Eligible participants were required to be (1) full-time male college students, (2) aged 18 years or older, and (3) willing to provide informed consent. Before accessing the survey, participants were presented with an electronic informed consent form describing the study purpose, procedures, voluntary nature of participation, confidentiality protections, and the right to withdraw at any time without penalty. Only those who selected “I agree to participate” were permitted to proceed to the questionnaire. Electronic informed consent was therefore obtained from all participants before data collection.

**Figure 1:**
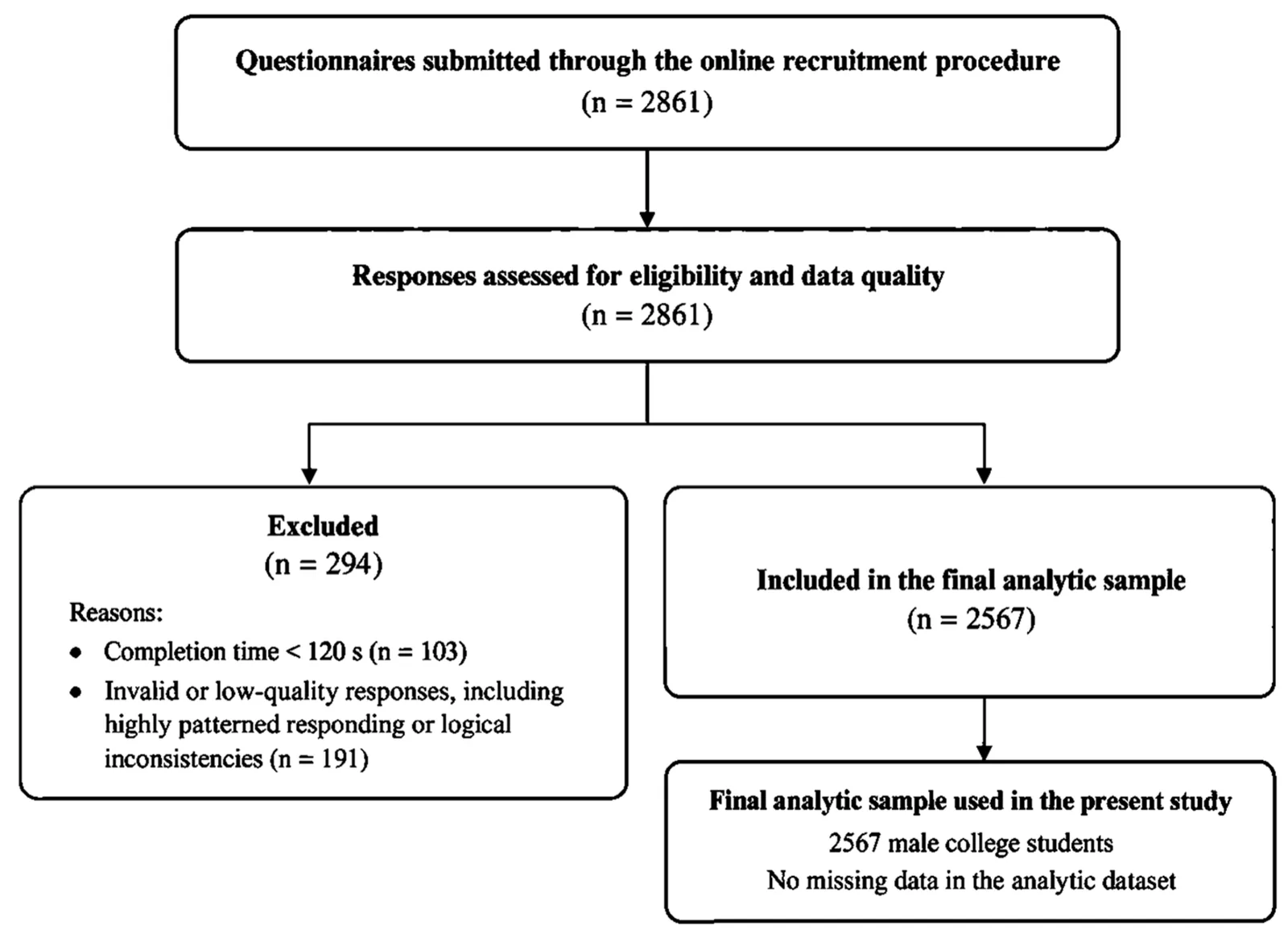
Flowchart of participant recruitment, eligibility assessment, exclusions, and final analytic sample.

To enhance data quality, responses were screened using prespecified criteria. Questionnaires completed in less than 120 s were excluded. The remaining responses were further screened for low-quality response patterns, including highly repetitive or invariant responding across consecutive items, incorrect responses to attention-check items, and obvious logical inconsistencies between related questionnaire responses. Responses identified as invalid according to these criteria were excluded from the final analytic sample. The participant recruitment, eligibility assessment, exclusion, and final sample-selection process is summarized in Figure 1. All study procedures were conducted in accordance with the relevant ethical requirements.

### 2.2. Measures

#### 2.2.1. Physical Activity

Physical activity (PA) was assessed using the revised Physical Activity Rating Scale-3 (PARS-3) developed by Liang [59], which has been widely used to assess exercise behavior in Chinese populations. The PARS-3 comprises three components assessing exercise intensity, duration, and frequency, each rated on a five-level behaviorally anchored scale. A composite PA score is calculated using the formula intensity × (duration − 1) × frequency, yielding a theoretical score ranging from 0 to 100, with higher scores indicating greater self-reported exercise volume [17]. Specifically, the duration score is transformed from 1–5 to 0–4 in the calculation, resulting in a minimum possible score of 0 (1 × 0 × 1) and a maximum score of 100 (5 × 4 × 5). The PARS-3 score represents a relative index of exercise volume rather than an estimate of moderate-to-vigorous physical activity minutes, MET-minutes, or adherence to WHO physical activity recommendations. In the present sample, the three PARS-3 components showed acceptable internal consistency (Cronbach’s α = 0.738). Given that intensity, duration, and frequency represent distinct but complementary components used to construct the PA index, this coefficient was treated as descriptive evidence of score consistency rather than as the sole indicator of measurement reliability. Detailed sample-specific factorial validity results are reported in Section 3.2.

#### 2.2.2. Non-Suicidal Self-Injury

NSSI was assessed using the 10 behavioral-frequency items from the Ottawa Self-Injury Inventory (OSI) [60]. The Chinese version of the OSI has demonstrated satisfactory reliability and validity and has been applied in Chinese adolescent and young adult populations [61,62]. The items assess distinct forms of self-injurious behavior during the previous 12 months using a four-point response scale (0 = never, 1 = once, 2 = 2–4 times, and 3 = five or more times). Item scores were summed to create an NSSI frequency index, with higher scores indicating greater frequency of self-injurious behavior. For prevalence and sensitivity analyses, any item score greater than 0 was additionally coded as past-year NSSI. All items were positively keyed and required no reverse scoring. Internal consistency was excellent in the present sample (Cronbach’s α = 0.940).

#### 2.2.3. Psychological Resilience

PR was measured using the 10-item Connor-Davidson Resilience Scale (CD-RISC-10) [63]. Each item is rated from 0 (not true at all) to 4 (true nearly all the time), and the items are summed to generate a total score, with higher scores indicating greater psychological resilience. All items are positively keyed and require no reverse scoring. The CD-RISC-10 has an established unidimensional structure and has been validated in Chinese populations [64]. Internal consistency was good in the present sample (Cronbach’s α = 0.813).

### 2.3. Data Analysis

All statistical analyses were conducted using SPSS 26.0 (IBM Corp., Armonk, NY, USA) and Mplus 8.3 (Muthén & Muthén, Los Angeles, CA, USA). Data completeness and response quality were examined before analysis, and no missing values were present in the final analytic sample (*n* = 2567). Descriptive statistics were calculated for all study variables, together with past-year NSSI prevalence and item-level endorsement rates. Pearson correlations were used to examine bivariate associations among PA, PR, and NSSI.

The sample-specific factorial validity of the study measures was evaluated using exploratory factor analysis (EFA). Sampling adequacy was assessed using the Kaiser–Meyer–Olkin (KMO) measure and Bartlett’s test of sphericity. Principal-axis factoring was used for factor extraction. Factor retention was evaluated with reference to eigenvalues greater than 1 and the theoretically established structure of each measure. Because a single factor was retained for each measure, no rotation was applied. Factor loadings, initial eigenvalues, extraction sums of squared loadings, and the percentage of variance explained were reported. Internal consistency was assessed using Cronbach’s α.

Because the NSSI frequency score was positively skewed and heteroskedasticity was detected, the primary regression models were estimated using HC3 heteroskedasticity-robust standard errors and 95% confidence intervals. Spearman rank-order correlations were conducted as a distribution-sensitive check of the bivariate findings, and logistic regression using binary past-year NSSI status served as an additional sensitivity analysis. Multicollinearity was evaluated using variance inflation factors (VIFs). Age, BMI, grade, only-child status, residence, and monthly living expense were included as covariates in the adjusted models.

To examine H2, PROCESS Model 4 was used to estimate the cross-sectional statistical indirect association of PA with NSSI through PR. Indirect effects were evaluated using 5000 bootstrap resamples and bias-corrected 95% confidence intervals. An indirect association was considered statistically significant when the bootstrap confidence interval did not include zero. Given the cross-sectional design, the analysis was interpreted as a statistical decomposition of associations rather than evidence of temporal mediation or causal transmission [65].

Separately, potential common method variance was evaluated using Harman’s single-factor test by entering the three PARS-3 components, ten CD-RISC-10 items, and ten NSSI items into an unrotated exploratory factor analysis. The proportion of variance accounted for by the first factor was examined using the conventional 50% heuristic. This procedure was treated as a diagnostic check rather than definitive evidence for the absence of common method bias.

To address H3, LPA was conducted using the three PARS-3 components and ten CD-RISC-10 items as continuous indicators after z-standardization. Models containing one through five profiles were estimated. Model selection was based on the Akaike Information Criterion (AIC), Bayesian Information Criterion (BIC), sample-size-adjusted BIC (aBIC), Lo–Mendell–Rubin likelihood ratio test (LMR-LRT), bootstrap likelihood ratio test (BLRT), entropy, profile size, parsimony, and substantive interpretability [66,67]. Within-profile covariances were fixed to zero, and indicator variances were constrained to equality across profiles in the primary specification. For the retained solution, average posterior probabilities and residual within-profile correlations were examined to assess classification quality and local independence. Differences in NSSI across the identified profiles were evaluated using one-way analysis of variance, with η^2^ and Cohen’s d reported as effect-size indices. All statistical tests were two-tailed, with *p* < 0.05 considered statistically significant.

## 3. Results

### 3.1. Participants’ Characteristics

A total of 2861 questionnaires were submitted online, of which 2567 met the prespecified validity criteria, yielding a valid-response proportion of 89.7% (2567/2861). No missing values were present in the retained analytic dataset. The mean age was 20.21 ± 1.33 years and mean BMI was 22.25 ± 2.86 kg/m^2^. Past-year NSSI (at least one endorsed behavior) was reported by 909 participants (35.41%).

Demographically, 36.30% of participants were freshmen, 30.40% sophomores, 19.30% juniors, and 14.00% seniors. Regarding family structure, 49.10% were only children, while 50.90% were non-only children. Regarding residential background, 42.20% of participants were from urban areas and 57.80% from rural areas. With respect to monthly living expenses, 14.80% reported less than 1000 RMB, 44.20% reported 1000–1999 RMB, 31.50% reported 2000–2999 RMB, and 9.50% reported 3000 RMB or above.

These demographic characteristics describe the participating sample only and should not be interpreted as evidence of representativeness. Detailed sample characteristics are presented in Table 1.

### 3.2. Measurement Validity and Reliability

Exploratory factor analyses were conducted to evaluate the sample-specific factorial structure of the three study measures. As summarized in Table 2, the data were suitable for factor analysis, with KMO values of 0.687 for PA, 0.919 for PR, and 0.969 for NSSI, and significant Bartlett’s tests of sphericity for all three measures (all *p* < 0.001). A one-factor structure was retained for each measure. Factor loadings ranged from 0.667 to 0.717 for the three PARS-3 components, from 0.519 to 0.575 for the CD-RISC-10 items, and from 0.752 to 0.801 for the NSSI items. The corresponding initial eigenvalues were 1.969, 3.727, and 6.479, while the extracted factors accounted for 48.50%, 30.31%, and 60.88% of the variance, respectively. Internal consistency was acceptable to excellent, with Cronbach’s α coefficients of 0.738 for PA, 0.813 for PR, and 0.940 for NSSI. Taken together, the factor-loading patterns, eigenvalues, explained variance, and reliability coefficients were generally consistent with the expected internal structure of the measures in the present sample.

### 3.3. Correlation Analysis

Descriptive statistics and Pearson correlations are shown in Table 3. Self-reported exercise volume was positively correlated with PR (r = 0.627, *p* < 0.01). Exercise volume and PR were negatively correlated with NSSI (r = −0.259 and −0.292, respectively; both *p* < 0.01). The corresponding Spearman correlations with NSSI were rho = −0.246 for exercise volume and rho = −0.266 for PR, supporting the same direction of association despite the skewed NSSI distribution. Harman’s single-factor test indicated that the first unrotated factor accounted for 32.70% of variance, suggesting that common method variance was unlikely to be explained by one dominant factor, although self-report common-method bias cannot be ruled out completely.

### 3.4. Covariate-Adjusted Regression and Indirect-Association Analyses

After adjustment for age, BMI, grade, only-child status, residence, and monthly living expenses, PA remained positively associated with PR (standardized β = 0.627, 95% CI [0.599, 0.655], *p* < 0.001). In the NSSI model, both PA (standardized β = −0.127, 95% CI [−0.168, −0.087], *p* < 0.001) and PR (standardized β = −0.211, 95% CI [−0.259, −0.164], *p* < 0.001) were inversely associated with NSSI. The adjusted NSSI model explained 9.8% of the variance (R^2^ = 0.098), indicating modest explanatory power. As shown in Table 4, the covariate-adjusted regression results were consistent across the focal predictors. In addition, the binary-NSSI sensitivity analysis yielded a similar pattern: for a 1-SD increase in PA and PR, the adjusted odds ratio were 0.73 for PA (95% CI [0.65, 0.82]) and 0.73 for PR (95% CI [0.65, 0.81]).

The bootstrap analysis was interpreted as a cross-sectional statistical indirect association rather than evidence of temporal mediation. As shown in Table 5, the total association between exercise volume and NSSI was −0.260 (95% CI [−0.290, −0.230]), while the direct association after inclusion of PR was −0.127 (95% CI [−0.165, −0.088]). The indirect association through PR was −0.133 (95% CI [−0.164, −0.102]), accounting for approximately 51% of the total model-based association. These results describe the specified cross-sectional statistical model only and do not establish that exercise volume precedes PR or that PR causally transmits an effect to NSSI.

### 3.5. Latent Profile Analyses

LPA was conducted using standardized indicators to prevent measurement units from determining profile separation. Fit statistics for one- through five-profile solutions are shown in Table 6. Although information criteria decreased with additional profiles, the two-profile solution retained the highest entropy (0.852), balanced estimated profile proportions (54.36% and 45.64%), highly significant LMR-LRT and BLRT comparisons against the one-profile model, and a parsimonious, stable interpretation. The five-profile LMR-LRT was not significant (*p* = 0.479). The final decision therefore did not rely on entropy alone but considered the complete fit pattern, classification quality, profile sizes, and substantive interpretability.

Classification diagnostics for the two-profile model were strong. Average posterior probabilities were 0.957 for the Low PA–Low PR profile and 0.954 for the High PA–High PR profile, corresponding to average classification uncertainty of approximately 4.3% and 4.6%, respectively. Based on participants’ most likely latent-profile membership, 1395 students (54.34%) were classified into the Low PA–Low PR profile and 1172 students (45.66%) into the High PA–High PR profile. Within modal profiles, 95% of absolute residual indicator correlations were below 0.14; the largest was 0.29. These values suggest that local dependence was limited but not absent, and this limitation is acknowledged. Indicator variances were constrained equal across profiles and within-profile covariances were fixed to zero in the primary specification.

The two profiles differed primarily in level: one showed consistently lower standardized PA and PR indicators, whereas the other showed consistently higher indicators. No stable discordant High PA–Low PR or Low PA–High PR profile emerged. This pattern may reflect the strong positive PA–PR correlation, the use of a single male-student sample, and the item-level measurement strategy. Accordingly, the profiles are interpreted as empirical positions along a coordinated PA–PR continuum rather than as qualitatively discrete clinical types.

**Figure 2:**
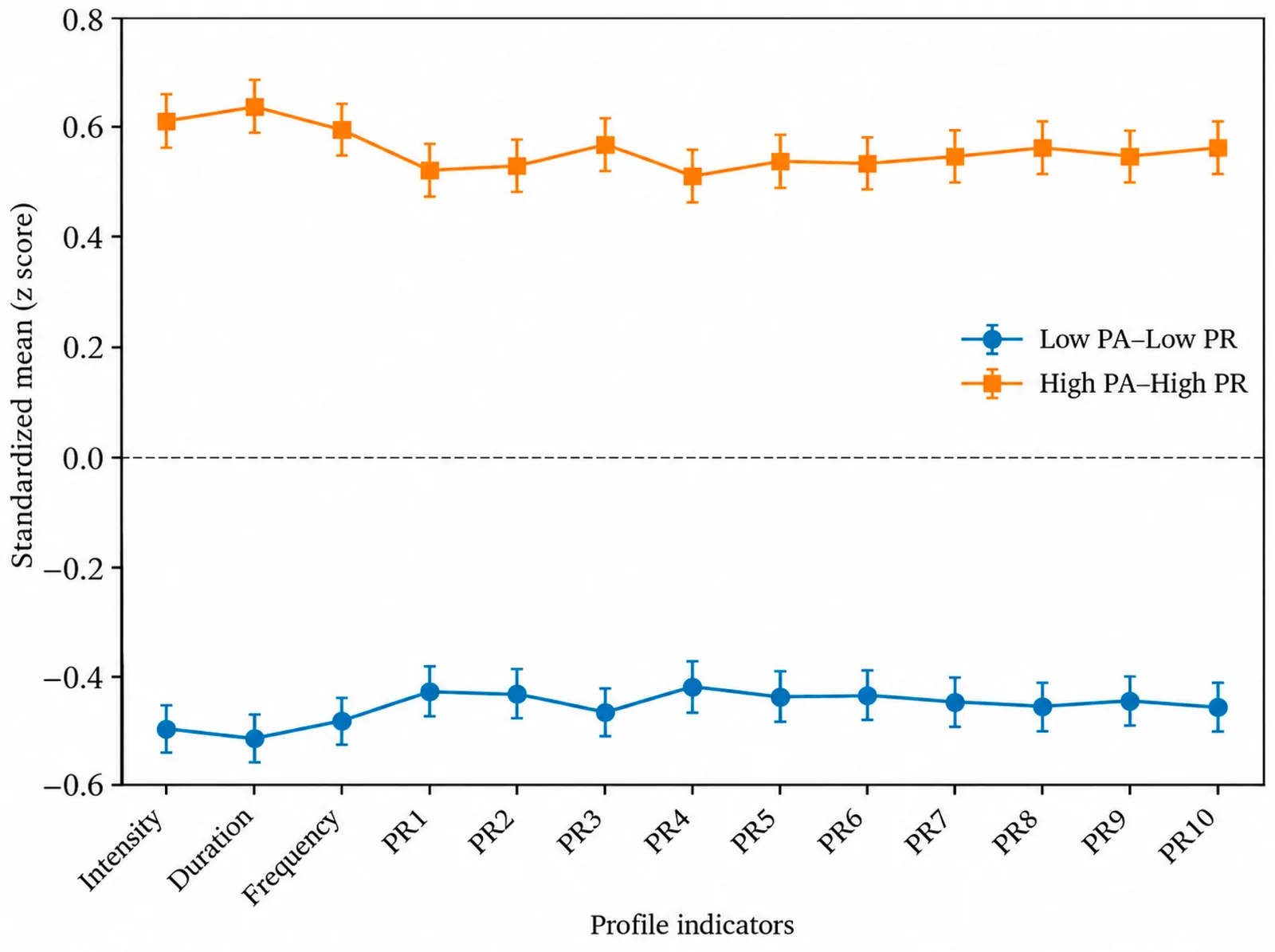
Standardized PA and PR profile indicators with 95% confidence intervals. The horizontal reference line at z = 0 represents the sample mean after standardization.

The profiles were therefore labeled Low PA–Low PR and High PA–High PR. Figure 2 presents standardized indicator means with 95% confidence intervals, making the relative separation and uncertainty directly visible.

NSSI differed significantly across the two latent profiles (Table 7). Participants classified into the Low PA–Low PR profile (*n* = 1395) reported higher NSSI scores (M = 7.54, SD = 9.38) than those classified into the High PA–High PR profile (*n* = 1172; M = 3.32, SD = 6.45). The one-way ANOVA indicated a significant difference in NSSI between the two profiles, F(1, 2565) = 169.77, *p* < 0.001, η^2^ = 0.062, with a standardized mean difference of Cohen’s d = 0.52. Age, BMI, grade, only-child status, residence, and monthly living expense did not differ significantly between the two profiles (all *p* > 0.05), reducing concern that the observed profile differences primarily reflected measured demographic characteristics.

## 4. Discussion

This study examined PA, PR, and NSSI using variable- and person-centered approaches. First, both PA and PR were negatively associated with NSSI after adjustment for all measured demographic characteristics. Second, PR statistically accounted for part of the cross-sectional association between PA and NSSI. Third, the LPA identified two predominantly level-based PA–PR profiles that differed significantly in NSSI scores.

### 4.1. Association Between Physical Activity and Non-Suicidal Self-Injury

The negative association between PA and NSSI supported H1. This association remained significant after adjustment for the measured demographic characteristics and was also supported by the Spearman sensitivity analysis. The similarity between the Pearson and Spearman estimates suggests that the observed association was not substantially affected by the distributional characteristics of NSSI. Prior studies have reported comparable inverse associations between PA and NSSI [18]. The current result extends this literature to male college students, who have received less focused attention in research on PA and NSSI.

Several factors may plausibly contribute to the observed association. For example, PA may be accompanied by greater social interaction, more structured daily routines, better emotion regulation, or stronger perceived competence [33,68,69,70,71]. These experiences may strengthen the behavioral and psychological resources that students draw on when managing distress. Their established relevance to NSSI suggests that PA may be associated with lower self-injury partly because it occurs within a wider pattern of adaptive daily regulation [72].

The stress-adaptation framework and conservation-of-resources theory provide useful perspectives for interpreting this pattern. The stress-adaptation framework emphasizes differences in the resources available to individuals when responding to stress, whereas conservation-of-resources theory emphasizes the acquisition and maintenance of resources that may support adaptation. Applied to the current findings, PA represents a behavioral context in which social connection, perceived competence, and structured coping may develop and accumulate, supporting more adaptive responses to stress [73,74].

The findings therefore position PA within a broader behavioral-resource account of NSSI. Its relevance may depend on the psychological and social experiences surrounding exercise participation, as well as the volume of exercise itself. Future longitudinal and intervention research can test this account by tracing changes in PA and NSSI over time and by directly assessing emotion regulation, social interaction, perceived competence, and daily structure as explanatory processes.

### 4.2. Statistical Indirect Association Through Psychological Resilience

The findings supported H2. PA was positively associated with PR, and PR was negatively associated with NSSI. The estimated indirect association between PA and NSSI through PR was statistically significant (indirect effect = −0.133, 95% bootstrap CI [−0.164, −0.102]), indicating that PR statistically accounted for part of the negative cross-sectional association between PA and NSSI. This pattern is broadly consistent with previous studies linking PA with stronger resilience and higher resilience with lower levels of NSSI [37,54,75].

The stress-adaptation framework and conservation-of-resources theory provide a plausible basis for interpreting this pattern. PA may occur alongside experiences such as goal attainment, perceived competence, social engagement, and successful coping with manageable demands, all of which may be associated with stronger psychological resources. PR, in turn, reflects an individual’s capacity to maintain or regain adaptive functioning under stress and may therefore be associated with a lower tendency to rely on maladaptive responses such as NSSI [76,77]. The observed indirect association is consistent with the possibility that PA and PR form part of a broader set of behavioral and psychological resources related to NSSI. Positive affect, self-efficacy, and emotion regulation offer specific pathways for examining how exercise-related experiences may contribute to the development of PR and its association with NSSI.

Importantly, the indirect association was estimated from variables measured at the same time point. The analysis therefore does not establish that PA precedes changes in PR or that PR subsequently influences NSSI. Reverse or reciprocal relationships also remain possible. For example, students with stronger PR may be more likely to engage in PA, whereas students with more frequent NSSI may report lower PA or PR. Longitudinal designs with repeated measurements, and ideally experimental studies, are needed to determine the temporal ordering of these associations.

From a practical perspective, the findings suggest that PA and PR may both be relevant when considering NSSI among male college students, but they should not be interpreted as sufficient intervention targets on the basis of the present data alone. Programs that combine opportunities for PA with approaches intended to strengthen resilience may warrant further evaluation, particularly in longitudinal or intervention studies. Given the modest proportion of variance in NSSI explained by the current model, future research should also consider other established correlates, including depressive symptoms, anxiety, trauma exposure, emotion dysregulation, family support, and social connectedness.

### 4.3. Differences in Non-Suicidal Self-Injury Across Latent Profiles

Building on the variable-centered findings, the present study used LPA to examine whether distinct joint patterns of PA and PR could be identified at the individual level. The two-profile solution was characterized by a low PA–low PR profile and a high PA–high PR profile. The two groups were relatively balanced in size, and the profile pattern showed that higher PA generally co-occurred with higher PR.

NSSI levels differed significantly between the two profiles, supporting H3. Participants in the low PA–low PR profile reported higher NSSI scores than those in the high PA–high PR profile. This pattern was consistent with the variable-centered findings, in which PA and PR were both negatively associated with NSSI. From a person-centered perspective, the results therefore suggest that lower levels of PA and PR tend to cluster within the same individuals and that this joint pattern is associated with higher NSSI.

At the same time, the profiles appeared to reflect primarily a low-to-high continuum rather than qualitatively distinct configurations. No discordant profiles, such as high PA–low PR or low PA–high PR, emerged. The relatively strong positive association between PA and PR in the present sample, which may have favored profiles in which both variables varied in the same direction. The indicator structure used in the LPA may also have contributed to this pattern, because the analysis combined three PA components with ten PR items. Although these indicators were standardized before profile estimation, the greater number of PR indicators may still have influenced the form of the resulting profiles.

The stress-adaptation framework and conservation-of-resources theory provide a useful basis for interpreting the profile differences. Students in the low PA–low PR profile may have fewer behavioral and psychological resources available when responding to stress, whereas those in the high PA–high PR profile may report a more favorable combination of these resources. However, the present data do not establish that these resource differences lead to changes in NSSI, nor do they identify the processes through which such differences might operate. Factors such as emotion regulation, social support, academic stress, and interpersonal difficulties may also contribute to the observed profile differences.

### 4.4. Practical Implications

The present findings have several potential implications for university health promotion and student support. First, the LPA identified a low PA–low PR profile and a high PA–high PR profile, with higher NSSI scores observed in the former group. Universities may therefore consider whether students showing relatively low levels of both PA and PR would benefit from broader health-promotion support. Such support could include accessible PA opportunities together with programs intended to strengthen resilience and coping resources.

Second, the results suggest potential value in closer coordination between physical and mental health services on university campuses. Because PA and PR were both associated with NSSI, and PR statistically accounted for part of the PA–NSSI association, student support programs may benefit from considering behavioral and psychological factors together. Collaboration between counseling services, health-promotion units, and physical education departments may provide a more integrated framework for supporting students’ overall well-being.

Finally, digital platforms may offer a complementary means of delivering health-promotion resources, particularly for students who are less likely to seek face-to-face support. Online PA programs, resilience-related resources, and psychological support may provide flexible and relatively accessible options for engagement.

### 4.5. Strengths and Limitations

This study has several strengths. First, the relatively large sample of male college students provided sufficient statistical power for both variable-centered and person-centered analyses. Second, the study combined conventional association analyses with latent profile analysis, allowing PA, PR, and NSSI to be examined from both average-level and individual-configuration perspectives. Third, several additional analyses were conducted in response to concerns regarding model robustness and reporting transparency. These included adjustment for all demographic variables available in the dataset, distribution-sensitive correlation analyses, common method bias assessment, multicollinearity and regression diagnostics, and more detailed reporting of LPA classification quality.

Several limitations should therefore be considered when interpreting the results. First, the cross-sectional design does not permit conclusions about temporal ordering among PA, PR, and NSSI. Although the statistical indirect association through PR was significant, the data cannot establish that PA precedes changes in PR or that PR subsequently influences NSSI. Reverse and reciprocal relationships remain plausible. For example, students with higher PR may be more likely to participate in PA, whereas students with more frequent NSSI may subsequently reduce their PA or report lower PR. The indirect association should therefore be interpreted as a cross-sectional statistical pattern. Longitudinal studies with repeated measurements and, where feasible, intervention designs are needed to clarify temporal relationships.

Second, all principal variables were assessed by self-report from the same participants at the same time point. Harman’s single-factor test showed that the first factor accounted for 32.70% of the variance and therefore did not indicate that one factor dominated the measurements. However, this test cannot rule out common method variance. Recall bias and social desirability may also have influenced the results, particularly because PA and NSSI are behaviors that may be over- or underreported. Future studies should consider objective measures of PA, such as accelerometry or wearable devices, and should incorporate temporally separated, multi-method, or multi-informant assessments where possible.

Third, the single-site sampling strategy limits the external generalizability of the findings. As described in the Methods, the baseline data were collected as part of a broader longitudinal project, and recruiting from one university helped maintain a stable cohort and support the feasibility of subsequent follow-up. In addition, because no female comparison group was included, no conclusions can be drawn regarding sex differences. Future studies should replicate these findings across multiple universities and regions, preferably using multisite, stratified, or probability-based sampling designs.

Fourth, the present analyses could adjust only for variables that were available in the original dataset. Age, BMI, grade, only-child status, residence, and monthly living expenses were included as covariates, but several potentially important confounders were not measured. These include depressive symptoms, anxiety, trauma history, emotion dysregulation, sleep quality, smoking, alcohol use, chronic illness, family support, social connectedness, personality characteristics, and more comprehensive indicators of socioeconomic status. Their omission may have contributed to residual confounding. Consistent with this concern, the regression model explained only 9.8% of the variance in NSSI. PA and PR should therefore be regarded as two correlates within a much broader set of psychological, behavioral, interpersonal, and contextual factors associated with NSSI.

Fifth, several limitations relate specifically to the LPA. The analysis included three PA components but ten PR items, producing an imbalance in the number of indicators representing the two constructs. Although the indicators were standardized before estimation, the larger number of PR indicators may still have influenced the formation of the profiles. In addition, the two-profile solution was characterized mainly by parallel low and high levels of PA and PR, while discordant patterns such as high PA–low PR or low PA–high PR were not identified. Thus, the profiles appear to represent primarily quantitative differences along a low-to-high continuum. Future studies should examine whether similar profiles emerge when more balanced construct-level indicators are used and should evaluate the stability and external validity of the profile solution in independent samples.

Finally, the sensitivity and diagnostic analyses supported the stability of the reported associations across alternative analytical specifications. Within the scope of the measured variables and the single-site observational design, the findings offer a transparent account of how PA, PR, and NSSI were related in this sample. These patterns also identify specific questions for longitudinal and multisite research, including the temporal ordering of the associations, the contribution of additional psychological and contextual factors, and their potential relevance to screening and prevention.

## 5. Conclusions

Among the male college students in this study, exercise volume and psychological resilience followed a clear pattern: students with lower levels of both reported more frequent NSSI. Psychological resilience statistically accounted for about half of the association between exercise volume and NSSI. The relationship between exercise and self-injury was therefore closely connected to students’ perceived capacity to adapt to stress.

## Figures and Tables

**Table 1:** Demographic Characteristics of Participants (*n* = 2567).

Variable	Category	*n*	%/M ± SD
Grade	Freshman	932	36.30%
	Sophomore	780	30.40%
	Junior	495	19.30%
	Senior	360	14.00%
Only Child	Yes	1260	49.10%
	No	1307	50.90%
Residence	Urban	1084	42.20%
	Rural	1483	57.80%
Monthly Expense	<1000	380	14.80%
	1000–1999	1135	44.20%
	2000–2999	808	31.50%
	≥3000	244	9.50%
Age			20.21 ± 1.33
BMI			22.25 ± 2.86

**Table 2:** Exploratory Factor Analysis and Internal Consistency of the Study Measures.

Measure	Factor Structure	Indicator	Factor Loading	Initial Eigenvalue	Extracted Variance (%)	KMO	Bartlett’s χ^2^ (df)	Cronbach’s α
PA	One factor	Intensity	0.705	1.969	48.5	0.687	1664.79 (3) ***	0.738
		Duration	0.667					
		Frequency	0.717					
PR	One factor	PR1	0.543	3.727	30.31	0.919	4988.19 (45) ***	0.813
		PR2	0.539					
		PR3	0.575					
		PR4	0.519					
		PR5	0.543					
		PR6	0.563					
		PR7	0.553					
		PR8	0.560					
		PR9	0.539					
		PR10	0.571					
NSSI	One factor	NSSI1	0.801	6.479	60.88	0.969	16,996.45 (45) ***	0.940
		NSSI2	0.775					
		NSSI3	0.774					
		NSSI4	0.789					
		NSSI5	0.780					
		NSSI6	0.752					
		NSSI7	0.770					
		NSSI8	0.774					
		NSSI9	0.796					
		NSSI10	0.790					

Note: *** *p* < 0.001.

**Table 3:** Means, Standard Deviations, and Correlations Among Variables.

Variable	M	SD	1	2	3
1. PA	26.635	28.038	1		
2. PR	19.603	9.205	0.627 **	1	
3. NSSI	5.627	8.449	−0.259 **	−0.292 **	1

Note: ** *p* < 0.01 (two-tailed).

**Table 4:** Covariate-Adjusted Regression Models with HC3 Robust 95% Confidence Intervals.

Predictor	Standardized β for PR (95% CI)	*p*	Standardized β for NSSI (95% CI)	*p*
PA	0.627 (0.599, 0.655)	<0.001	−0.127 (−0.168, −0.087)	<0.001
PR	-	-	−0.211 (−0.259, −0.164)	<0.001
Age	−0.002 (−0.033, 0.029)	0.903	0.024 (−0.013, 0.061)	0.205
BMI	−0.012 (−0.043, 0.018)	0.426	0.001 (−0.035, 0.037)	0.950
Only child (No vs. Yes)	0.029 (−0.031, 0.089)	0.345	0.010 (−0.063, 0.084)	0.781
Residence (Rural vs. Urban)	−0.040 (−0.102, 0.021)	0.198	−0.006 (−0.081, 0.069)	0.873
Grade: Sophomore vs. Freshman	−0.003 (−0.078, 0.071)	0.928	−0.056 (−0.147, 0.036)	0.232
Grade: Junior vs. Freshman	0.068 (−0.017, 0.153)	0.118	−0.048 (−0.150, 0.054)	0.353
Grade: Senior vs. Freshman	0.020 (−0.078, 0.118)	0.688	0.039 (−0.081, 0.158)	0.524
Expense: 1000–1999 vs. <1000	−0.040 (−0.133, 0.054)	0.405	0.106 (−0.004, 0.216)	0.058
Expense: 2000–2999 vs. <1000	−0.058 (−0.156, 0.041)	0.251	0.063 (−0.052, 0.178)	0.282
Expense: ≥3000 vs. <1000	−0.179 (−0.305, −0.053)	0.005	0.105 (−0.055, 0.264)	0.199
Model R^2^	0.397		0.098	

**Table 5:** Bootstrap Cross-Sectional Direct and Indirect Associations (5000 Resamples).

Pathway	Effect	Boot SE	95% CI		Proportion of Effect
			LLCI	ULCI	
Total	−0.260	0.016	−0.290	−0.230	100%
PA → NSSI	−0.127	0.020	−0.165	−0.088	49%
PA → PR → NSSI	−0.133	0.016	−0.164	−0.102	51%

**Table 6:** Latent Profile Analysis Fit Indices.

Profiles	AIC	BIC	aBIC	LMR-LRT	BLRT (*p*)	Entropy	*n* (%) by Category
1	120,275.34	120,427.45	120,344.84	/	/	/	/
2	113,606.83	113,840.85	113,713.76	<0.001	<0.001	0.852	54.36/45.64
3	112,611.01	112,926.93	112,755.36	<0.001	<0.001	0.780	35.33/38.86/25.81
4	112,270.07	112,667.91	112,451.85	<0.001	<0.001	0.758	31.09/14.80/26.41/27.70
5	112,113.86	112,593.60	112,333.07	0.479	<0.001	0.711	12.86/25.55/18.56/18.66/24.37

**Table 7:** NSSI Differences Between the Two Modal Latent Profiles.

Outcome	Low PA–Low PR (*n* = 1395), M ± SD	High PA–High PR (*n* = 1172), M ± SD	F(1, 2565)	*p*	η^2^	Cohen’s d
NSSI	7.54 ± 9.38	3.32 ± 6.45	169.77	<0.001	0.062	0.52

## Data Availability

The data are not publicly available because they contain sensitive information concerning non-suicidal self-injury and other psychological and behavioral characteristics of human participants, and unrestricted public disclosure could compromise participant confidentiality. De-identified data supporting the findings of this study are available from the corresponding author upon reasonable request, provided that the proposed use is consistent with the informed consent and confidentiality requirements of the study.

## Abbreviations

The following abbreviations are used in this manuscript:

PA	Physical Activity/self-reported exercise volume (PARS-3 index)
PR	Psychological Resilience
NSSI	Non-Suicidal Self-Injury
LPA	Latent Profile Analysis
PARS-3	Physical Activity Rating Scale-3
CD-RISC-10	10-item Connor–Davidson Resilience Scale
OSI	Ottawa Self-Injury Inventory
BMI	Body Mass Index
COR	Conservation of Resources
AIC	Akaike Information Criterion
BIC	Bayesian Information Criterion
aBIC	Sample-Size Adjusted Bayesian Information Criterion
LMR-LRT	Lo–Mendell–Rubin Adjusted Likelihood Ratio Test
BLRT	Bootstrap Likelihood Ratio Test
LLCI	Lower Limit Confidence Interval
ULCI	Upper Limit Confidence Interval
SPSS	Statistical Package for the Social Sciences
